# Pathogenesis and clinicohistopathological caractheristics of melanoacanthoma: A systematic review

**DOI:** 10.4317/jced.52860

**Published:** 2016-07-01

**Authors:** Elena Cantudo-Sanagustín, Aída Gutiérrez-Corrales, Manuel Vigo-Martínez, María-Ángeles Serrera-Figallo, Daniel Torres-Lagares, José-Luis Gutiérrez-Pérez

**Affiliations:** 1 Master in Oral Surgery; 2 Medical Doctor. Diplomate in Dental Surgery. Lecture in Oral Medicine. University of Seville; 3 Professor of Oral Surgery. Co-Head of Master in Oral Surgery. University of Seville

## Abstract

**Introduction:**

The melanoacanthoma is a rare benign pigmented tumor, characterized by a fast radial growth and clinical behavior similar to melanoma. Color changes in oral mucosa and dermis are consequence of increased melanocyte activity as response to an irritant factor. There is a vast phenotypic variety. It is difficult to distinguish between a benign pigmented lesion and a melanoma at its early stage. Due to its clinical relevance is crucial to diagnose possible malignancy of the lesions.

**Objectives:**

The aim of this article is to conduct a systematic review of all published articles, as well as update and evaluate etiologic factors and clinicopathological features.

**Material and Methods:**

We carried out a search in the Medline database (PubMed) using the key words “oral melanoacanthoma” AND “oral melanoacanthosis” AND “oral melanoepithelioma”. Inclusion criteria were all published articles since its discovery. Demographic data, histological features and immunohistochemical findings were extracted from the full articles.

**Results:**

A total of 56 articles were analysed. 114 injuries drawn from these articles were studied, a total of 115 injuries with our contribution case. The 74.78% of authors claim a reactive pathogenesis. The average age of lesión appearance is 34.79 years, with an age range of 5-87 years. There is a predominance of the female sex in solitary phenotype 3: 2 and a ratio of women to men 5: 3 if it is multifocal phenotype. Bilateral phenotype is slight higher in women of 2: 1.

**Conclusions:**

Histopathological analysis of the lesión is vital to diagnose malignancy. Therefore, any heterogeneous, pigmented lesion with irregular edges, raised surface, fast growth and abrupt appearance should be biopsied. More emphasis on the potential irritants should also be put to improve the quality of life of our patients and to reduce morbidity of melanoacanthoma, as well as, several similar clinical behavior disease.

** Key words:**Melanoacanthoma, oral cáncer, diagnosis.

## Introduction

Melanoacanthoma was first described by Bloch in 1926 as melanoepitheliomoa. In 1960, Mishima and Pinkus introduced the term melanoacanthoma to clarify the term melanoepithelioma type 1 and 2 previously described by Bloch in 1927 (1). The term melanoacanthoma corresponds to Bloch`s melanoepithelioma type 1. First lesion in the oral mucosa was presented by Tomey and Dorey in the Maxilofacial and Oral Pathology Congress of the American Academy, in 1978. According to this revision, Schneider *et al.* described their first case in 1981 (2).

Since then, solitary and, less frequent, multiple lesions have been described in the oral mucosa with a total number of 115 cases to the date in our search.

Melanoacanthoma is a rare benign mixed epitelial tumor, characterized by the mucocutanean pigmentation with dendritics melanocytes dispersed among the epithelium with acanthosis areas, espongiosis on melanyne presency. The presence of inflammatory infiltration of linfocitic and eosinophils is a common find (3,4).

The high of incidence is between the third or fourth decade, it shows higher prevalence in black race and women although some cases were reported in Caucasia race.

**Figure 1:**
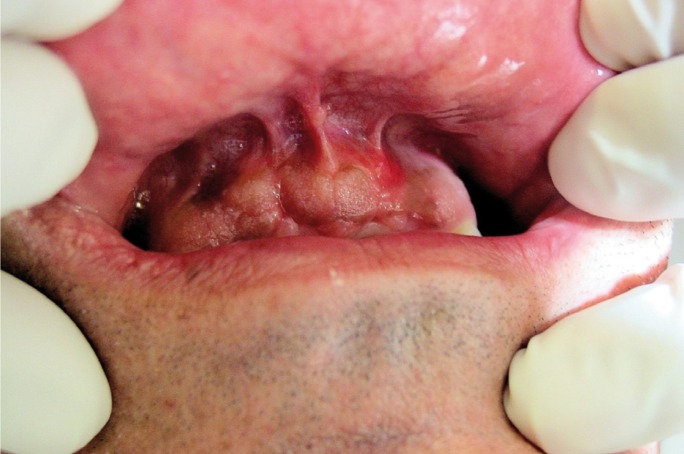
Histological images of the pyogenic granuloma showing an appearance similar to granulation tissue. The histological type of the pyogenic granuloma is non-lobular capillary hemangioma. Arrow heads label blood vessels surrounded by connective tissue.

Lesions may occur as isolated or multiple, plained or raised, with well defined or diffuse edges and the color ranges from dark brown to black. There have been described multiple cases and others with bilateral lesion (Fig. 1). Melanoacanthoma lesions ca be asymptomatic or develop with pain, burning or itching. Its etiology is related to irritative or traumatic factors (5).

Cutaneous melanoacanthoma are more likely to appear in head, neck and chest and less frequently in the eyelids or lips. Intraoral lesions are usually asymptomatic and preferentially located in the buccal mucosa (47.54%), palate (18.03%), lips (11.47%) and gum (5.6%) (6).

While cutaneous melanoacanthoma never dissapear, oral melanoacanthoma can regress after the elimination of irritating factor or after being biopsied. Cutaneous variant occurs mainly in fair-skinned adults while the oral melanoacanthoma has a predilection for blacks and younger patients (7).

Radial growth it is a high potential pathognomonic sign, it can mask a subyacent melanoma (3).

These characteristics have been studied with electronic microscopy, and several tests had been used like: inmunoprecipitation test with the aim of analize patient´s serum to search antibody antimelanoma, inmunofluorescence to look for present anthygens on melanoma lesion: inmunohistochemical studies have demostrated melanocitic reactivity of the melanocytes which reside on the basal, parabasal and cellular espinous strate for the marquer HMB-45; the protein S-100 serves as marquer of the presence of melanocitics dendritics cells, very useful for its diagnosis confirmation, as the marquer Melanin- A also is used with this purpose (7).

**Figure 2:**
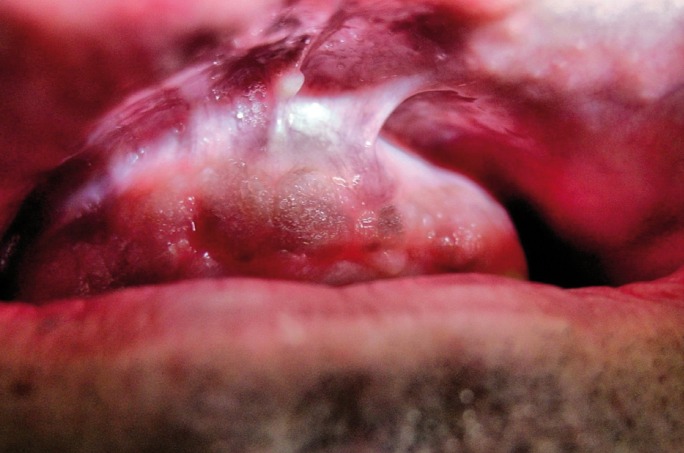
Same lesion after a follow up of 10 years. It keeps stable.

Quirurgical exéresis shows a great ratio of success without recurrences (Fig. 2). It offers the advantage of preserve the borders of the lesion for a histhopathologic analisis. Sometimes, even after the incisional biopsy, an involution of the lesion is observed with high frequency. Other ways of treatment are laser ablation with Argon, crioterapy, curettage and the topic application of Flourouracil 5% (8).

The aim of the present article is to make a systematic review of all the published cases, as to actualize and evaluate ethiologic factors and it clinicopathologic characteristics.

## Material and Methods

A systematic, computerized database search was conducted using the National Center for Biotechnology Information (NCBI) to search MEDLINE (Pubmed). The search was conducted using the following MeSHterms:” “oral melanoacanthoma” AND “oral melanoacanthosis” AND “oral melanoepithelioma”.

For the initial selection, we selected all articles published since melanoacanthoma. Demographic data, histological characterestics and immunohistochemical findings were taken from the full text. From the literature a total of 59 articles, in relation with mela-noacanthoma, were obtained, three of those were exclude after complete reading. We evaluated 56 articles. A total of 115 patients, including the case presented by our team, were diagnosed.

**Figure 3:**
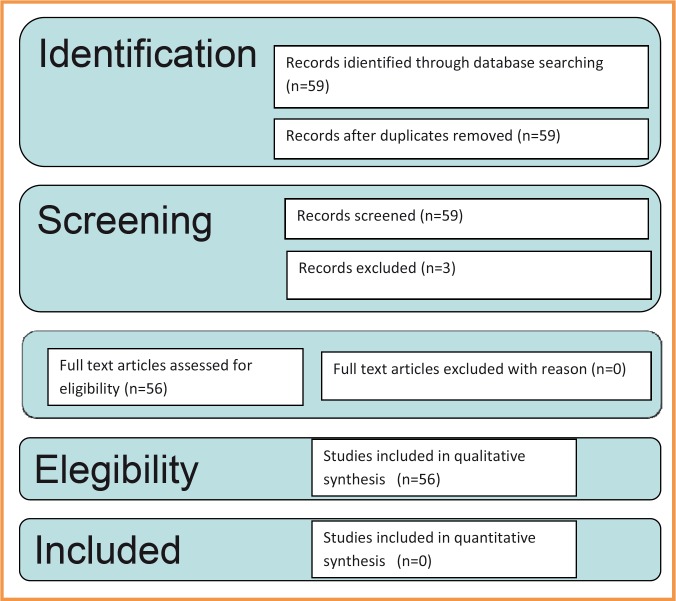
Prisma Flow Diagram: different stages of the search in a systematic review.

Figure 3 describes, in a flow diagram, search phases of our systematic review.

## Results

The review of the literature shows that oral melanoacanthoma affects patients aged between 5 and 87 years, with a mean age of 34.79 years. There is higher prevalence in women, 54.4% versus 38.4% in men.

The ratio female-male is 3: 2. There is a predominance of the female sex in solitary phenotype 3: 2 and when the multifocal phenotype is the ratio female-male is 5: 3. In the bilateral phenotype is slight higher in women, 2: 1.

The solitary phenotype appears more frequently (18.26% of cases) than the multifocal phenotype (13.91% of cases).

The locations from highest to lowest frequency are buccal mucosa 33.9%, 13.04% palate, 5.22% lips, 13.91% alveolar mucosa including retromolar área and lingual mucosa, 3.48% tongue, 4.35% back, 3.48% abdomen, 3.48% scapula, 3.48% ear, 3.48% eyelid, 2.6% leg, 2.6% buttock, 1.74% neck, 1.74% floors mouth, 1.74% nose, 1.74% armpit, chest 0.87%, 0.87% vermilion lipstick, hip 0.87%, 0.87% base of the penis, arms 0.87% 0.87% temporal region, 0.87% submental region, 0.87% scrotum groin area, 0.87% preauricular area ,0.87% shoulder and 0.87% forehead.

The most frequent presentation is blackish brown in 40.8% of cases, followed by bluish black 3.2%, 1.6% reddish brown and grey 0.8%.

It has a predilection for black race (37.39%) followed by caucasian (19.13%), latin American (3.47%) and Asians (3.47%). 77.42% of the authors assert that the etiology is related to irritative factors.

 Table 1,  Table 1 continue shows all cases submitted for analysis and the results of our systematic review.

**Table 1:**
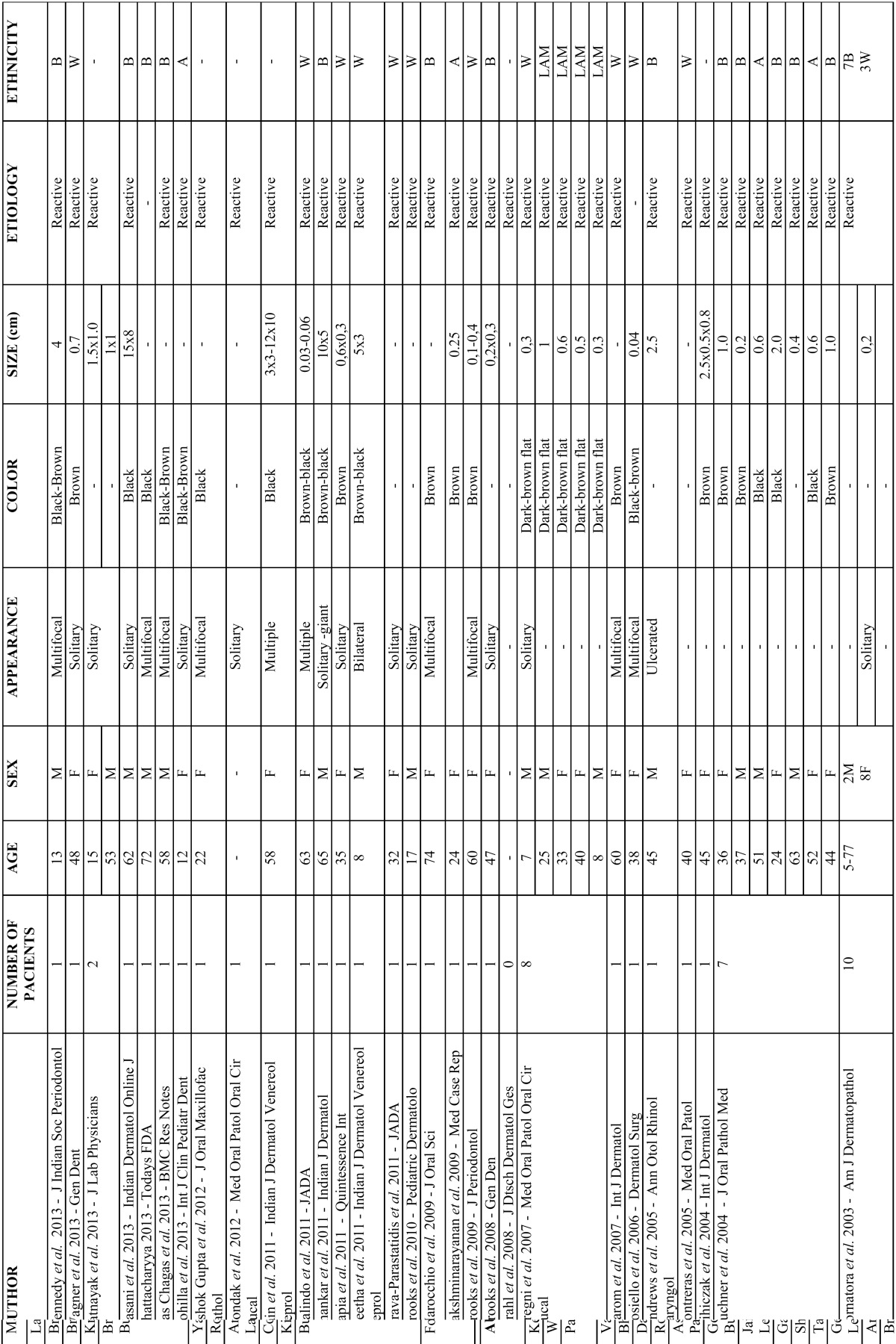
Described cases of melanoacanthoma found in literature search.

**Table 1 continue:**
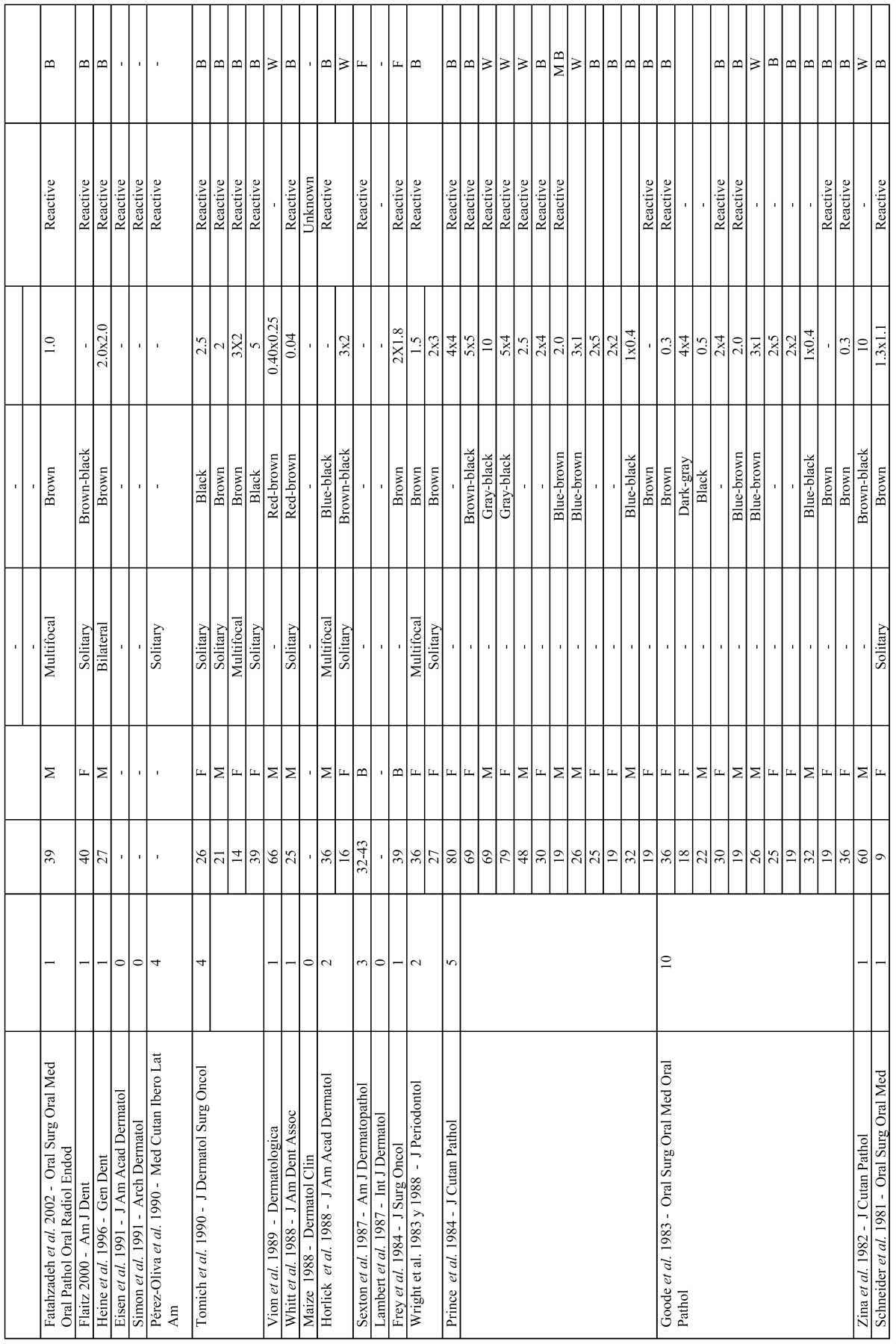
Described cases of melanoacanthoma found in literature search.

**Table 1 continue-1:**
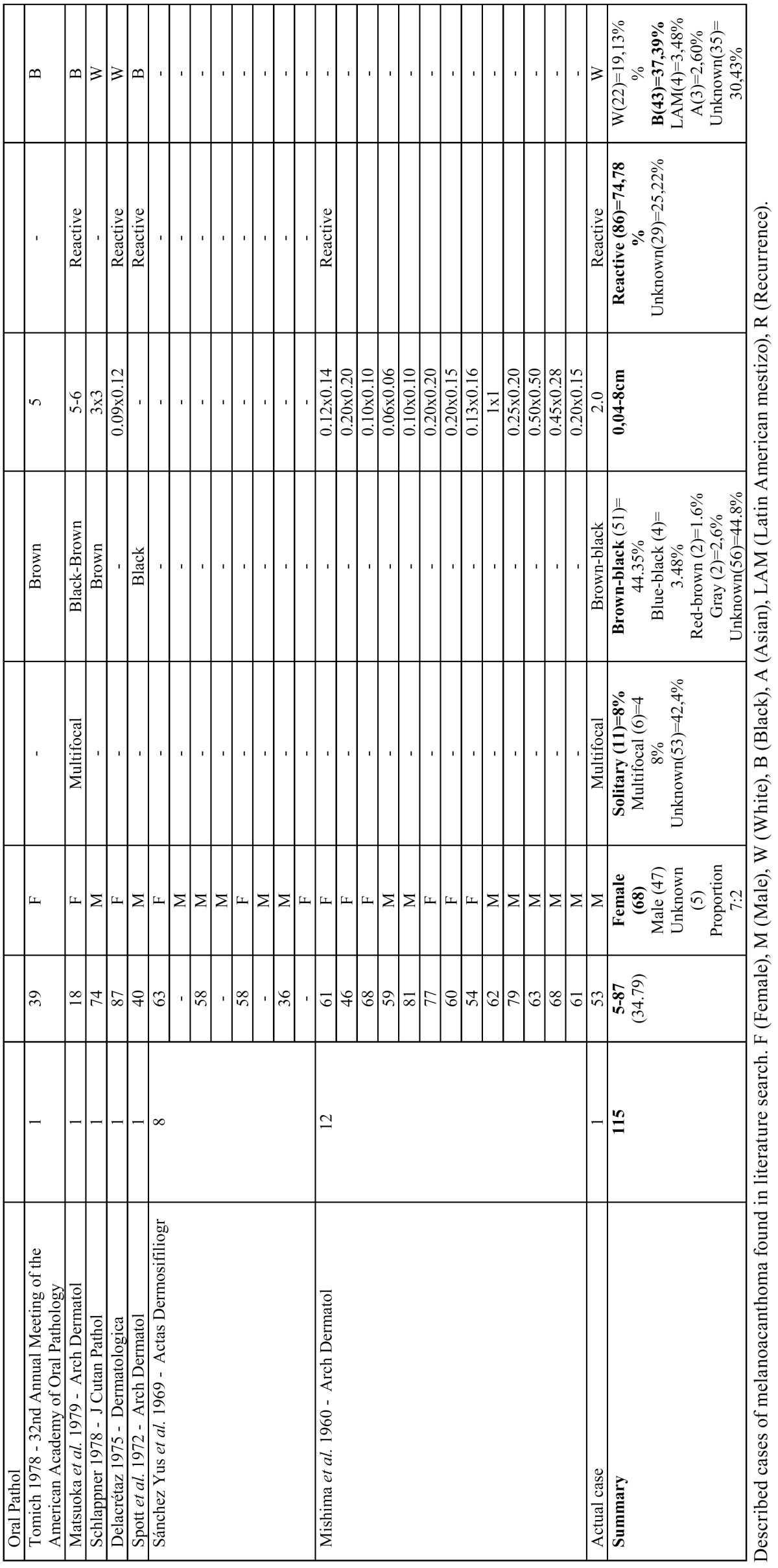
Described cases of melanoacanthoma found in literature search.

## Discussion

The variability of phenotypic expression justifies the controversy of the classification of this entity. There are different phenotypes with multiple expressions: cutaneous or oral melanoacanthoma, painful or asymptomatic, fast growing or stable, multiple or solitary melanoacanthomas; as well as different locations and histopathologic features.

Zemtsov *et al.* consider that oral melanoacanthoma is a tumor wrongly named and defined it as an unusual proliferation of dendritic melanocytes mucositis in the epidermis (9). Horlick, propose the term mucosal melanotic macula for the reactive type of this lesion (10).

The etiology is still unclear, but most of the authors associate it with a continuing traumatic process that stimulate melanocytic activity (3,5,11,12).

Most of the injuries are related to trauma and its appearance varies from weeks to months. They even dissapear after eliminating irritants or biopsy. This fact makes the reactive etiology stronger.

The reactive etiology of melanoacanthoma and other pigmented lesions may be associated with chronic contact with petroleum derivatives, such as sodium lauryl sulfate, nitropheno, phentolphthalein, clorophenol, phenylenediamine sulfate, cocamidopropyl betaine or amine fluoride. These components are found in toothpastes and mouthwashes and they act as irritants causing morphological changes. Pathology 100% of lesions supports this, and it is related to oral and cutaneous melanoma phenotype (13-15).

Most of the the authors observe the ocurrence of these lesions in trauma areas of bruxism patients, lesions matching the occlusal plane or very prominent cusps of molars and premolars. Likewise, lesions have also been described in patients with a recent dental restoration, which entails a soft tissue trauma during the adaptation period (16,17).

Silver amalgam fillings have also been described as etiological factors that may cause pigmentation and changes in the epithelium (17). There is much controversy with this restorative material. Many countries, like Germany and the US, have already forbbided its use in dental therapy due to its corrosion and risk of toxicity, while other countries like Spain argue that this material has been used for hundred of years without an apparent risk.

In 2007 Yarom *et al.* describe as etiologic factors ill-fitting removible prothesis, patients treated for chronic asthma, constant bite of the cheeks, hydrogen peroxide mouthwashes and nonspecific chronic trauma (18,19).

There are also described in the literature lesions suddenly appeared after implants surgery or associated with ferrous lactate chronic treatment for iron-deficiency anemia.

Zemtsov *et al.* proposed to their patients removing toothpastes and mouthwashes containing hydrogen peroxide resulting in the spontaneous resolution of melanoacanthoma. They show that the most common irritant are mercury and petroleum derivatives as cinnamic aldehyde, in toothpastes, which may cause allergic contact dermatitis (8).

Toothpastes with abrassive components, such as, calcium phosphate or calcium carbonate, act as irritant factors which produce tissue reaction after cronic contact (20).

Brooks *et al.* found that there are alterations of superficial dermis in cutaneous phenotype, but they didn´t observed fast growth or spontaneous resolution as it does in oral melanoacanthoma (21,22).

Galindo *et al.*, along with other authors, argue that melacantoma does not need any specific treatment or follow up because there have not been reported cases of malignancy with features of dysplasia or atypia (6).

However, we found in the literatura (Zina, in 1982), a case of simple hydroacanthoma with a malignant transformation into a porocarcinoma. This extremely rare tumor classification is highly complex due to its histopathological similarities with other lesion and degenerative changes of any lesion with age. The relationship between melanocytes and keratinocytes is very similar to the realtion observed in the melanoacanthoma (23). This rare lesion was named Bort-Jadasshon intraepidermal epithelioma and it matches with the terms described by Bloch in 1927 and Mishima in 1960 when melanoacanthoma was called “non-cutaneous benign melanoepitelioma nevoid” (24,25).

Simon believes for there are three variants of melanocytic seborrheic keratosis: irritant, non-irritating and nested variants. He considers that oral melanoacanthoma is an irritant seborrheic keratosis and he suggests the term melanoacanthoma should be removed (12).

Concerning relations between melanoacanthoma and seborrheic keratosis it should be noted that the only difference between the two processes is that in melanoacanthoma can be found many melanocytes at every level of tumor epidermis, while in seborrheic keratoses, melanocytes are not increased and they can only be found in basal area (26). Authors like Sanchez Yus and Simon Huarte concluded that both tumors are the same entity and they should not be separated. The electron microscope, shows that the distribution and arrangement of melanocytes are notably different in melanoacantomas compared to seborrheic keratoses. Melanocytes are small with intense mitotic activity, melanin granules are present in the cytoplasm and several basal keratinocytes proliferate (27).

Clinical appearance similar to other pigmented lesions, family history, drug use or systemic drugs, hormones, heavy metals and changes in the morphological pattern are important for the differential diagnosis (11). Some pathological entities described in  Table 2,  Table 2 continue should be included.

**Table 2:**
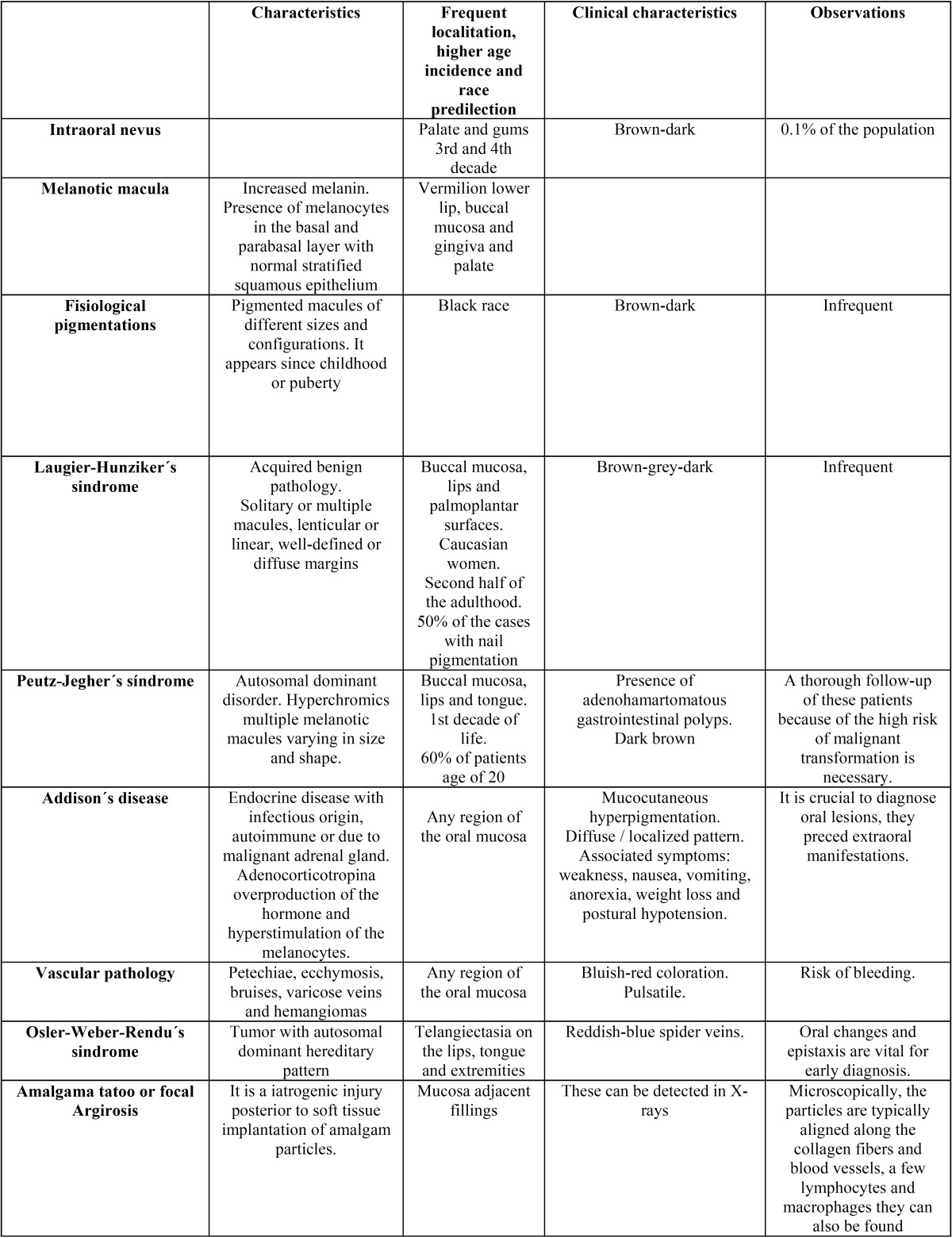
Differential diagnosis of various pathologies.

**Table 2 continue:**
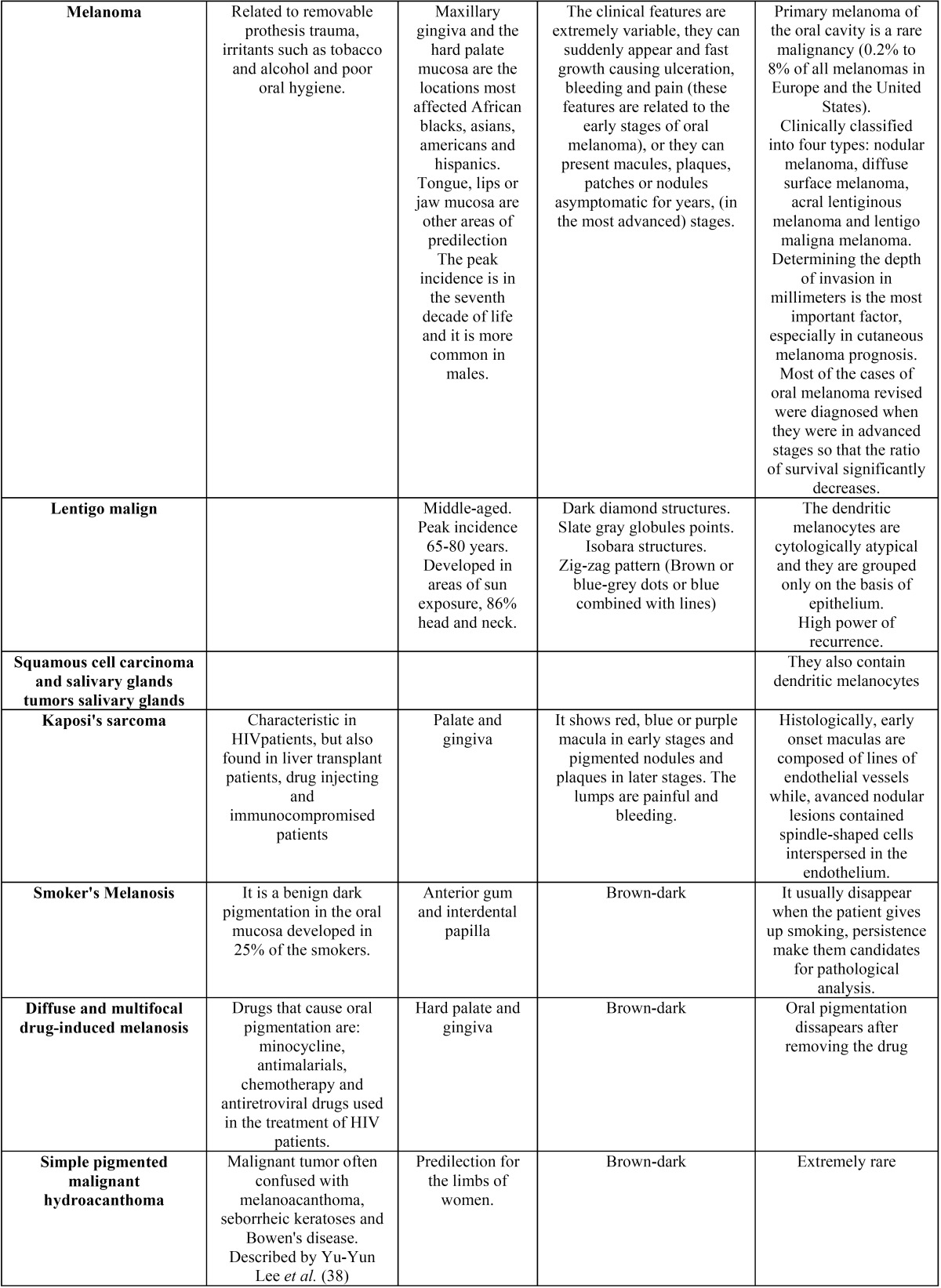
Differential diagnosis of various pathologies.

Back to melanoacanthoma histology, this is a pigmented tumor that exhibits great morphological variability. Pathological characteristics show stratified squamous epithelium with proliferation of melanocytes and melanin presence in the basal layer and suprabasal layers without invading the underlying connective tissue, prone to the central keratinization (endoqueratinización). The predominant cell pattern prickly keratinocytes or basal differentiation is present in different areas. Melanocytes have extensive dendritic processes and striking areas of acanthosis (27).

The presence of inflammatory infiltrate is found in the great majority of melanoacanthomas. The block in the transference of melanin from melanocytes to keratinocytes is the nature of this entity. Alteration in the normal pattern and speed differentiation of keratinocytes alters cell characteristic of keratinocytes surface which inhibits pigment donation.

The etiological hypotheses of reactive origin pigmented lesion is supported by the frequent presence of inflammatory infiltrate of lymphocytes.

Langerhans cells are present in every Malpighian layer except from the basal layer. These cells are related to proliferation control of keratinocytes. Therefore, the study of Langerhans cells is interesting due to its disposition at every layers (27).

## Conclusions

The histopathological analysis of the melanoacanthoma suspicious lesion is crucial to rule out malignancy, as it may hide a subyacent oral melanoma. Any heterogenous pigmented lesion with irregular borders, raised surface, fast growth and sharp appearance should be biopsied. Since over 75% of reported cases indicate an irritating background, more emphasis must be put on the control of them.This can improve the quality of life of our patients and reduce morbidity of numerous pathologies.
